# Antitumor effect and mechanism of FZD7 polypeptide vaccine

**DOI:** 10.3389/fonc.2022.925495

**Published:** 2022-10-05

**Authors:** Zhongke Hua, Yu Han, Kan Liu, Hua Yang, Cai Zhou, Fengyi Chen, Shenglan Nie, Mengqing Li, Qinyao Yu, Yunpeng Wei, Christina C. N. Wu, Xiaomei Wang

**Affiliations:** ^1^ International Cancer Center, Shenzhen Key Lab of Synthetic Biology, Shenzhen University Health Science Center, Shenzhen University, Shenzhen, China; ^2^ Moores Cancer Center, University of California, San Diego, La Jolla, CA, United States

**Keywords:** cancer prevention, cancer immunology, FZD7, TLR7 agonist, breast cancer

## Abstract

The resistant cells that proliferate after radiotherapy and chemotherapy are primarily tumor stem cells with high stem marker expression, and their presence is the primary cause of tumor dispersion. The Wnt signaling receptor Frizzled family receptor 7 (FZD7) is linked to the maintenance of stem cell features as well as cancer progression. Frizzled-7 (FZD7), a key receptor for Wnt/-catenin signaling, is overexpressed in TNBC, suggesting that it could be a viable target for cancer therapy. We employed bioinformatics to find the best-scoring peptide, chemically synthesized FZD7 epitope antigen, and binding toll-like receptor 7 agonists (T7). Under GMP conditions, peptides for vaccines were produced and purified (>95%). *In vivo* and vitro tests were used to assess tumor cell inhibition. *In vitro*, the FZD7-T7 vaccination can boost the maturity of BMDC cells considerably. In mice, the FZD7 - T7 vaccine elicited the greatest immunological response. Significant tumor development inhibition was seen in BALB/c mice treated with FZD7 - T7 in prevention experiments (P < 0.01). Multiple cytokines that promote cellular immune responses, such as interferon (IFN)-γ (P < 0.05), interleukin (IL)-12 (P < 0.05), and IL-2 (P < 0.01), were shown to be considerably elevated in mice inoculated with FZD7- T7. Furthermore, we evaluated safety concerns in terms of vaccine composition to aid in the creation of successful next-generation vaccines. In conclusion, the FZD7-T7 vaccine can activate the immune response *in vivo* and *in vitro*, and play a role in tumor suppression. Our findings reveal a unique tumor-suppressive role for the FZD7 peptide in TNBC.

## Introduction

Cancer is a major public health issue that affects people all over the world, and it is the second-highest cause of death in the United States (1). Breast cancer (BC) is the second leading cause of cancer-related death in women (2, 3). To avoid immune surveillance and eradicate anti-tumor immune responses, cancer cells have acquired a range of features, including deficiencies in antigen presentation mechanisms, activation of negative regulatory pathways, and recruitment of immunosuppressive cell populations (4). Tumor treatment is becoming more diverse, with chemotherapy, surgery, and radiotherapy as the mainstays, and immunotherapy and other new treatments gaining traction (5, 6). Currently, there are many treatments for tumor immunity, including tumor vaccine, cell adoptive immunotherapy, cytokine therapy, gene therapy, etc (7, 8). Wnt/β-catenin signaling has been implicated in different stages of mammary gland development and is important for mammary oncogenesis (9). Studies have demonstrated that activation of Wnt/β-catenin signaling is preferentially found in TNBC and is associated with a poor clinical outcome (10, 11). It was recently discovered that FZD7 was up-regulated in TNBC and TNBC-derived cell lines (12) and that FZD7 modulated TNBC cell tumorigenesis through the canonical Wnt signaling pathway (13). Wnt proteins are secreted glycoproteins that bind to the N-terminal extra-cellular cysteine-rich domain of the Frizzled (FZD) receptor family. FZD receptor binds to WNT protein and can activate WNT pathway. FZD7 plays an important role in stem cell biology and cancer development and progression (14). In addition, it has been demonstrated that siRNA knockdown of FZD7, the anti-FZD7 antibody (15) displayed anti-cancer activity *in vitro* and *in vivo* mainly due to the inhibition of the canonical Wnt signaling pathway. Therefore, targeted inhibition of FZD7 represents a rational and promising new approach to cancer therapy (16, 17). Polypeptides will be a novel form of vaccination since they are immunogens that cause effector cell immunological responses *in vivo* (18, 19). At the moment, polypeptide vaccines are a hot topic in vaccine research (20). Peptide vaccines are made using chemical peptide synthesis technology and are based on the amino acid sequence of a pathogen antigen gene epitope that is known or predicted (21). The immunological dominant region of FZD7 was used in this study. In conjunction with a Toll-like receptor 7 (T7) agonist, we developed and synthesized its linear fragments. Significant tumor suppression was achieved prophylactically in FZD7- T7-treated BABL/c mice. FZD7 - T7 produced much greater immune responses and decreased tumor growth in BABL/c mice without causing significant systemic harm, according to our findings.

## Materials and methods

### Epitope prediction

The protein sequences of FZD7 were obtained from the National Center for Biotechnology Information (NCBI; http://www.ncbi.nlm.nih.gov). The T cell epitope prediction tool from the Immune Epitope Database and Analysis resource (www.IEDB.org). The sequences of FZD7 were analyzed. The peptide sequence was FZD7: DAGLEVHQFYPLVKVQCSPELRFFLCSMYAPVCTVLDQAI

### Experimental animals and cell lines

Female BALB/c mice were purchased from Guangzhou Medical Laboratory Animal Center, Guangdong Province, China. The mice were finally used for about 5-6 weeks. All animal treatments and experimental protocols in this study were approved by the Laboratory Animal Center and Laboratory Animal Ethics Committee of Shenzhen University School of Medicine (Permit No. AEWC-2019003). All mice were housed under a 12-h light/dark cycle, at 23 ± 1°C, with 39%–43% relative humidity. Water and food were provided ad libitum. 4T1 cells (The 4T1 cell line represents TNBC, and over 1,000 studies have reported on the 4T1 cell line model.) were maintained in Dulbecco’s Modified Eagle’s Medium (DMEM) with 10% fetal bovine serum (FBS) and 100 U/mL penicillin-streptomycin, and all of these reagents were obtained from Hyclone Laboratories, Inc. (South Logan, UT, USA).

### Vaccine preparation

The FZD7 peptide was synthesized at ChinaPeptides Co., Ltd. (Shanghai, China).

The adjuvant, Toll-like receptor (TLR) 7 agonist, was supplied by Biodragon Immunotech Inc. (Beijing, China) and was mixed with FZD7 peptide at a 1:1 volume.

### Mouse immunization and establishing tumors

Female BALB/c mice (6–10 per group) were vaccinated with 50μg of different vaccines *via* intramuscular (i.m.) injection on days 0, 7, 14). Seven days after the final vaccination, the mice were then injected (s.c.) with 4T1 cells (1 × 10^5^) into the lower flank of the right-back and the tumor size was measured periodically with calipers. After cell inoculation, the tumor volume was measured every 2 days. Tumor size was calculated using the formula: 0.5×length×width^2^ (cm^3^). Mice were euthanized when the tumor size was > 1.5 cm^3^.

### Enzyme-linked immunosorbent assay (ELISA)

Sera samples were collected 3–5 days after three doses of vaccine had been administered. Blood was taken from the orbital vein, and serum was obtained by centrifuging the blood at 3000 rpm for 20 min at 4°C. An ELISA for multiple cytokines in peripheral blood was performed using a Ready SET-GO! ELISA kit according to the instructions provided by the manufacturer (eBioscience, Thermo Fisher Scientific).

### Pathological examination

Hematoxylin-eosin (H&E) staining was performed using a Hematoxylin and Eosin Staining Kit (Beyotime). Organs of mice and cancer tissues were fixed in formaldehyde (10%) for 48 h, followed by dehydration, permeabilization, wax dipping, paraffin embedding, and cutting into 3 μm sections. After staining with hematoxylin (300 sec), sections were stained with eosin solution for 30 sec. Next, sections were dehydrated and mounted using neutral gum. Images were captured by microscopy (Olympus CX23; Olympus Corporation, Tokyo, Japan).

### Immunofluorescence staining

Immunofluorescence staining for CD3 was performed on formalin-fixed paraffin-embedded tumor tissue sections. Sections were deparaffinized using xylol and rehydrated using decreasing concentrations of ethanol (100, 95, and 70%) followed by a short wash in distilled water. Heat-induced antigen retrieval was performed in a citrate-based buffer in a 700 W microwave. Sections were blocked and permeabilized in PBS containing 10% horse serum and 0.1% Triton X-100 for 1 h at room temperature followed by overnight incubation at 4°C with either rabbit anti-CD3 monoclonal antibody (1:500, ab16669; Abcam, Cambridge, UK). After three 5 min washes in PBS, sections were incubated for 2 h at room temperature with AlexaFluor 488 (goat anti-rabbit, 1:500, ab16669; Abcam). Immunofluorescence staining for mucin 2 was performed on Carnoy’s solution (60% absolute ethanol, 30% chloroform, and 10% acetic acid) fixed and paraffin-embedded colon tissue sections using rabbit anti-mucin 2 polyclonal primary antibody (1:100; antibodies-online GmbH, Aachen, Germany) and DyLight^®^594 conjugated donkey anti-rabbit polyclonal secondary antibody (1:250; Abcam). Nuclear counterstaining was performed with a mounting medium containing DAPI (F6057; Sigma-Aldrich). Images were acquired using a Nikon Eclipse E400 microscope (Nikon, Tokyo, Japan) with an Olympus DP73 camera (Olympus, Tokyo, Japan) and excellence Entry software (Olympus).

### TUNEL staining

TUNEL assays for tumor tissues were conducted using an *In Situ* Cell Death Detection Kit, TMR red (Roche, USA) according to the kit instructions.

Dewax formalin-fixed tissue sections according to standard procedures. Place the slide(s) in a plastic jar containing 200 ml 0.1 M Citrate buffer, pH 6.0. Apply 750 W (high) microwave irradiation for 1 min. Cool rapidly by immediately adding 80 ml double dist-water (+20 to +25°C). Transfer the slide(s) into PBS (+20 to +25°C). Immerse the slide(s) for 30 min at +15 to +25°C in Tris-HCl, 0.1 M pH 7.5, containing 3% BSA and 20% normal bovine serum. Rinse the slide(s) twice with PBS at +15 to +25°C. Let excess fluid drain off. Add 50 μL of TUNEL reaction mixture to the section. Note: For the negative control add 50 μL Label solution. Incubate for 60 min at +37°C in a humidified atmosphere in the dark. Rinse slide(s) three times in PBS for 5 min each. Evaluate the section under a fluorescence microscope.

### Monitoring for adverse events

Laboratory monitoring for adverse events including the body weight, section of viscera tissue, and Viscera index of experimental mice before tumor inoculation was measured to evaluate the safety of this animal model.

### Statistical analysis

We used GraphPad Prism 8.0 for statistical analysis. For longevity, Kaplan-Meier survival was used, and p-values were calculated using the logarithmic rank test. To compare the two groups, students’ T-tests were performed. A one-way analysis of variance (ANOVA) was performed using Duncan’s test to compare multiple groups. P <0.05 was considered statistically significant.

## Results

### Bioassay identified FZD7 as a therapeutic target

**Figure 1:**
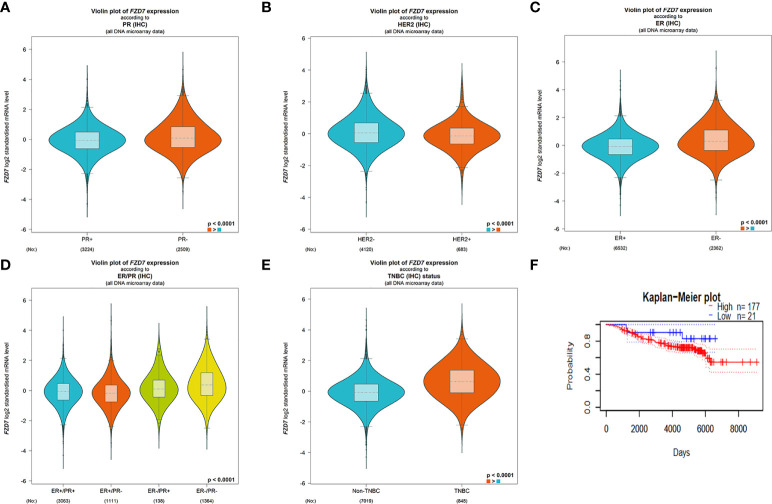
BC-GenexMiner V4.3 and PrognoScan: Boxplot assessment of FZD7 gene expression and survival analysis based on clinical parameters of breast cancer patients. **(A)** PR. **(B)** HER-2. **(C)** ER. **(D)** ER/PR. **(E)** triple-negative status. **(F)** overall survival.

The expression of the FZD7 gene in several clinical parameters in breast cancer patients was evaluated using BC-GenexMiner V4.3 software. There was a significant difference between the PR positive and PR negative groups (**Figure 1A**, P <0.0001). FZD7 gene expression was higher in PATIENTS with HER2-negative breast cancer (**Figure 1B**, P <0.0001). FZD7 gene expression was higher in patients with ER-negative breast cancer (**Figure 1C**, P <0.0001). The expression level of FZD7 in ER-/PR-breast cancer patients was significantly higher than that in other groups in (**Figure 1D**, P <0.0001). In addition, FZD7 was significantly higher in patients with triple-negative breast cancer than in patients with non-triple-negative breast cancer (**Figure 1E**, P <0.0001). Survival curves were drawn using survival analysis software PrognoScan, and data and GSE7390 showed increased FZD7 expression associated with poorer overall survival (**Figure 1F**). Low expression of FZD7 is associated with better clinical prognostic parameters and clinical survival for breast cancer. According to bioinformatics analysis, FZD7 plays a significant role in the occurrence and development of triple-negative breast cancer (TNBC), suggesting that it is a good therapeutic target.

### Vaccine preparation and injection

**Figure 2:**
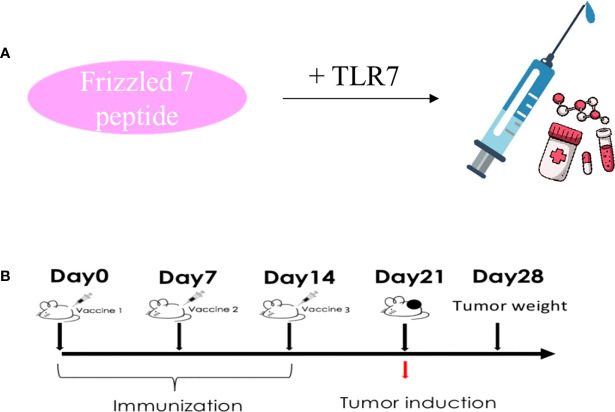
Protocol design route. **(A)** Vaccine preparation process diagram. **(B)** Immunological administration and tumorigenesis schedule.

First of all, we first through the table to determine the antigen epitope prediction program, a sequence of FZD7: DAGLEVHQFYPLVKVQCSPELRFFLCSMYAPVCTVLDQAI. Vaccine peptides (>95%) were synthesized and purified according to Good Manufacturing Practice (GMP). T7 is a mature immune vaccine adjuvant used in combination with polypeptides. The FZD7 antigenic polypeptide is coupled to T7 (**Figure 2A**). About the timing of vaccination. It is very important to choose the best vaccination time during cancer treatment. Vaccination schedules, including primary and booster vaccinations, are related to the effectiveness and duration of anti-tumor T cell responses. Therefore, we determined the preventive administration program for BALB/C mice before the experiment. The immune adjuvant used in the experiment had the advantages of fewer immunizing injections (once every 7 days, three times in total) and low antigen dosage (antigenic polypeptide FZD7 50 micrograms) (**Figure 2B**). The vaccine is water-soluble, uses the muscular immune pathway, and does not require the complex emulsification process of Freund’s adjuvants.

#### Immunogenicity of the vaccine *in vitro*

**Figure 3:**
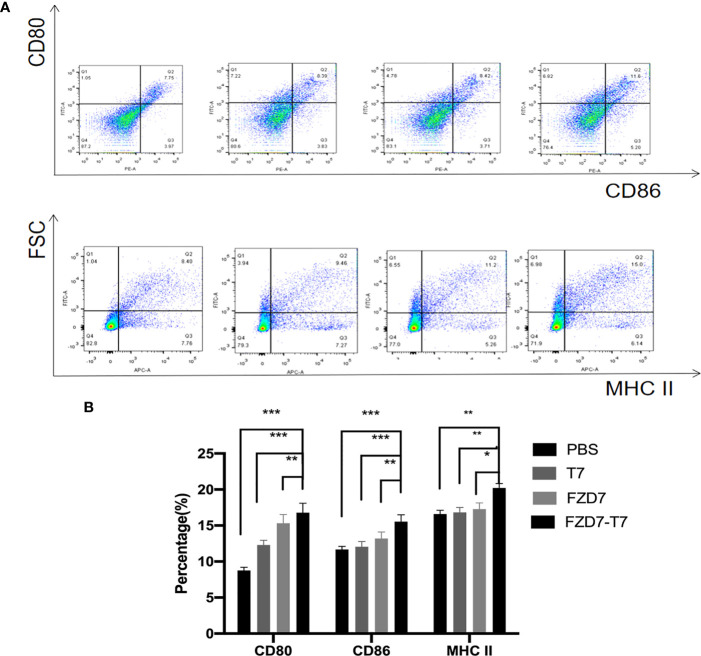
Expression of CD80, CD86, and MHC II on the surface of bone marrow-derived dendritic cells detected by flow cytometry. **(A)** Flow cytometry images. **(B)** Quantitative analysis of flow cytometry results. *P < 0.05, **P < 0.01, ***P < 0.001.

Mature DC plays an important role in tumor immunity because it can activate both T cells and B cells to release cytokines. We used the peptides identified and synthesized in the figure above to determine *in vitro* whether the peptides could more effectively stimulate DC cell maturation. Therefore, peptides and adjuvants were co-cultured with immature DC, and surface maturation markers CD80, CD86, and MHC II were analyzed by flow cytometry (**Figure 3A**). FZD7-T7 stimulated DC cells significantly increased CD80 and CD86 expression (***P < 0.001 vs. PBS&T7), and MHC II expression was significantly increased (**P < 0.01 vs. PBS&T7) in FZD7-T7 group (**Figure 3B**). The above data indicate that FZD7-T7 stimulation can promote DC maturation. The immunogenicity of the combined vaccine was verified *in vitro*.

#### Immunogenicity of the vaccine *in vivo*

**Figure 4:**
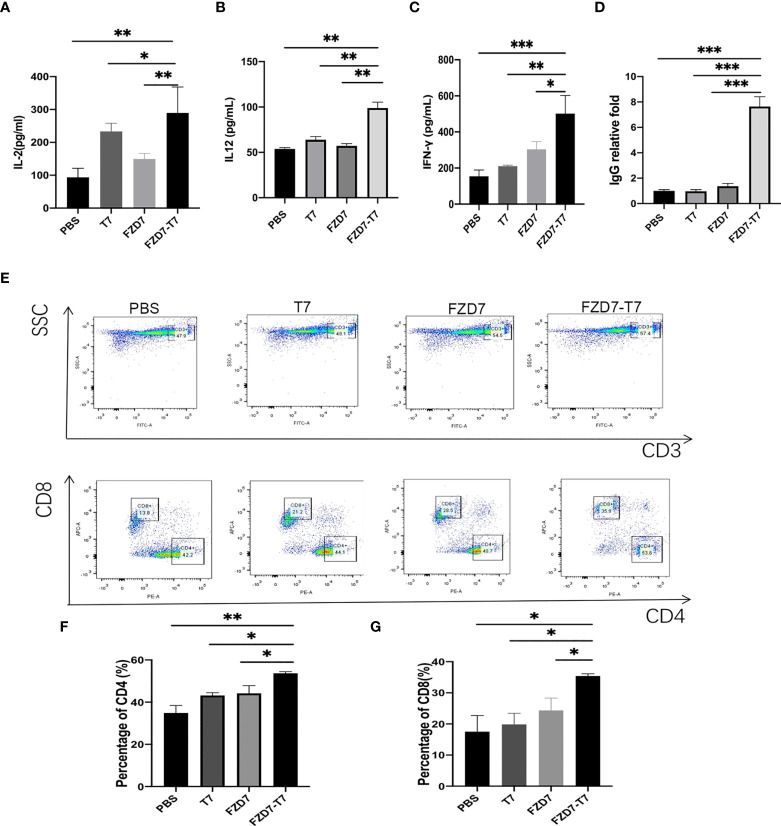
Assess cytokine, antibody, and T cell responses *in vivo.* **(A)** IL-2. **(B)** IL12. **(C)** IFN-γ. **(D)** ELISA of specific antibody content of FZD7 peptide after different vaccine stimulation. **(E)** Flow cytometric lymphocyte isolation. **(F)** The ratio of CD3^+^/CD4^+^ T cells was detected. **(G)** CD3^+^/CD8^+^ T cells ratio (*P < 0.05, **P <0.01, ***P < 0.001).

The immunogenicity of the FZD7-T7 combined vaccine was studied. Sera were collected and the levels of interferon (IFN)-γ, interleukin IL-12, and IL-2 cytokines were detected. The ELISA results (**Figures 4A–C**) indicated that the levels of secreted IFN-γ, IL-12, and IL-2 were significantly higher in the FZD7- T7 vaccine group compared with other groups (*P < 0.05, **P < 0.01, ***P < 0.001). After evaluating the immunogenicity of the vaccine, we investigated whether the vaccine stimulated humoral immunity and observed a significant increase in FZD7 IgG (**Figure 4D**) antibody levels in mice immunized with the FZD7-T7 vaccine (all three groups ***P < 0.001). In T cell subsets (**Figures 4E–G**), CD4^+^ and CD8^+^ T cell cancers play an important role. FZD7-T7 not only increases the proportion of CD3^+^/CD8^+^ T cells in spleen cells (all three groups *P < 0.05), but also increases the proportion of CD3^+^/CD4^+^ T cells(Compared with T7&FZD7, *P < 0.05; Compared with PBS, **P < 0.01), suggesting that vaccines can activate T cell responses.

### Evaluate the antitumor effect of the FZD7-T7 vaccine

**Figure 5:**
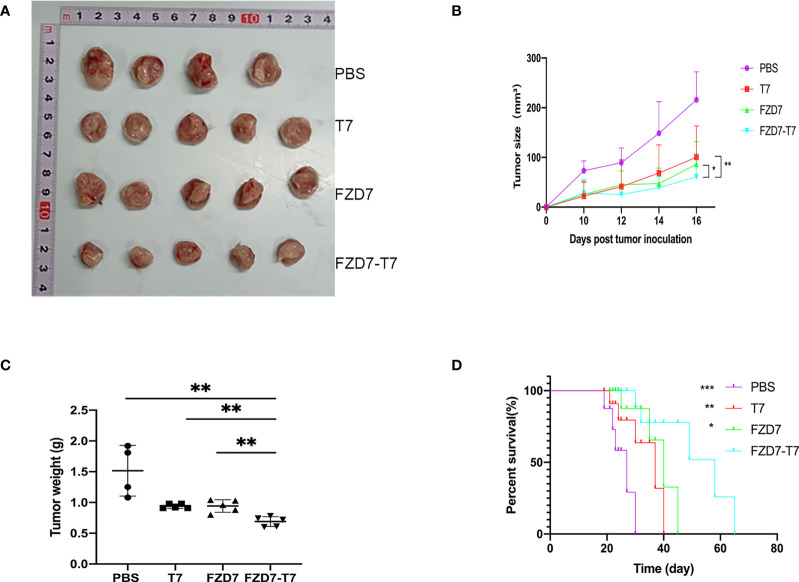
Anti-tumor effects of the FZD7 vaccine. **(A)** Image of tumor size. **(B)** Tumor volume (mm3) was assessed every two days. **(C)** tumor weight. **(D)** Survival curve of mice after tumor growth. *P < 0.05, **P < 0.01, ***P < 0.001.

We evaluated the antitumor efficacy of the combination vaccine by using a tumor-bearing assay. According to the immune administration plan, we immunized BALB/C mice with PBS as a negative control 3 times and then inoculated 10^5^ 4T1 tumor cells in the left leg subcutaneously. Tumor size was recorded 10 days later. Tumor volume was recorded every two days (**Figure 5B**). Tumor volume =1/2 x length x width^2^. On day 16, the mice were sacrificed by carbon dioxide asphyxia and cervical dislocation. Tumor specimens were collected, photographed, and weighed (**Figures 5A, C**). Reduced tumor growth rate was observed in all treatment groups (compared with FZD7, *P < 0.05; **P < 0.01); And FZD7-T7 vaccine group had the best anti-tumor effect (all three groups **P < 0.01). This supports the hypothesis that combination vaccine therapy produces an immune response that inhibits tumor growth. Based on the above data, we wondered whether the combination vaccine could also prolong the survival time of 4T1-bearing mice. All mice in the PBS group died 30 days after tumor growth. The survival time of mice immunized with FZD7-T7 was significantly prolonged (compared with PBS, ***P < 0.001; Compared with T7, **P < 0.01; Compared with FZD7, *P < 0.05; (**Figure 5D**). These results suggest that FZD7-T7 combined vaccine has a good therapeutic effect on the 4T1 tumor model. Therefore, based on the above analysis of tumor inhibition rate, we hope to conduct further research on FZD7 and T7 combination vaccine.

### Analyze tumor microenvironment

**Figure 6:**
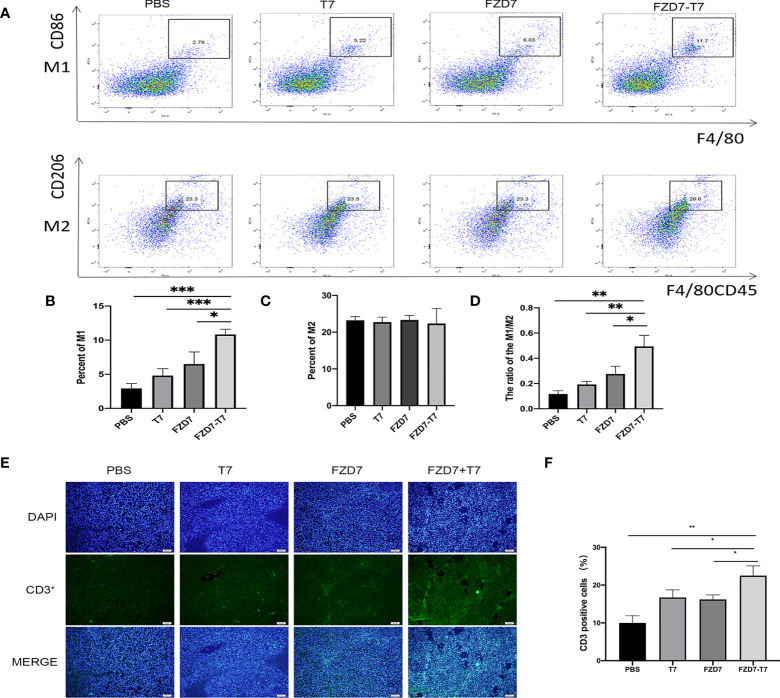
Analyze Tumor microenvironment. **(A)** Flow cytometry of TAM cells. **(B)** percentage of M1 cells. **(C)** percentage of M2 cells. **(D)** The ratio of M1/M2. **(E)** CD3+ immunofluorescence sections of tumor tissue. **(F)** Quantitative analysis of the proportion of CD3+ T cells in tumor tissues. *P < 0.05, **P < 0.01, ***P < 0.001.

After the isolation and extraction of tumor-associated macrophages TAM from mouse tumors (**Figure 6A**), we found that the number of CD86 + F4/80 +, namely M1 TAM, increased in mice immunized with the FZD7-T7 vaccine compared with the control group (**Figure 6B**), while, There was no difference in the number of CD206 + F4/80CD45 + (M2 TAM) cells (**Figure 6C**). By measuring the ratio of M1-positive cells to M2-positive cells(**Figure 6D**), macrophages were significantly upregulated to the M1 phenotype compared with the control (compared with FZD7, *P < 0.05; Compared with T7, **P < 0.01; And PBS, ***P < 0.001).

(M1: Monitoring tumor lesions and anti-tumor effect. M2: Inhibits immune response and promotes tumor effect). Histopathological examination showed that tumor immune CD3 infiltration was increased in FZD7-T7 mice compared with other groups, suggesting that T cells may be effector cells mediating tumor regression (**Figures 6E, F**), indicating immune cell recruitment triggered by the FZD-T7 vaccine.

### Analysis of tumor tissue apoptosis

**Figure 7:**
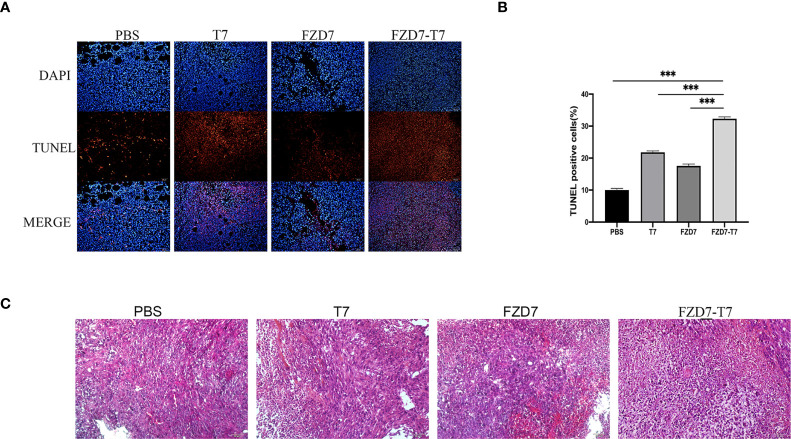
Analyze Tumor tissue. **(A, B)** TUNEL positive percentage of tumor tissue measured by TUNEL assay. **(C)** HE staining of tumor tissue sections. ***P < 0.001.

TUNEL stained cells were categorized as “TUNEL positive” or “TUNEL negative” under the fluorescent microscope. We examined the apoptosis of tumor cells by TUNEL staining and found a significant increase in the percentage of apoptotic tumor cells in tumor tissues immunized with the FZD7-T7 vaccine. TUNEL analysis further confirmed that FZD7-T7 induced extensive apoptosis of tumor cells (**Figures 7A, B**). Moreover, histological analyses of tumor sections indicate that FZD7-T7 triggered extensive tumor necrosis, and FZD7 and T7 could also induce somewhat tumor necrosis as compared with the control (**Figure 7C**).

### Toxicity studies

**Figure 8:**
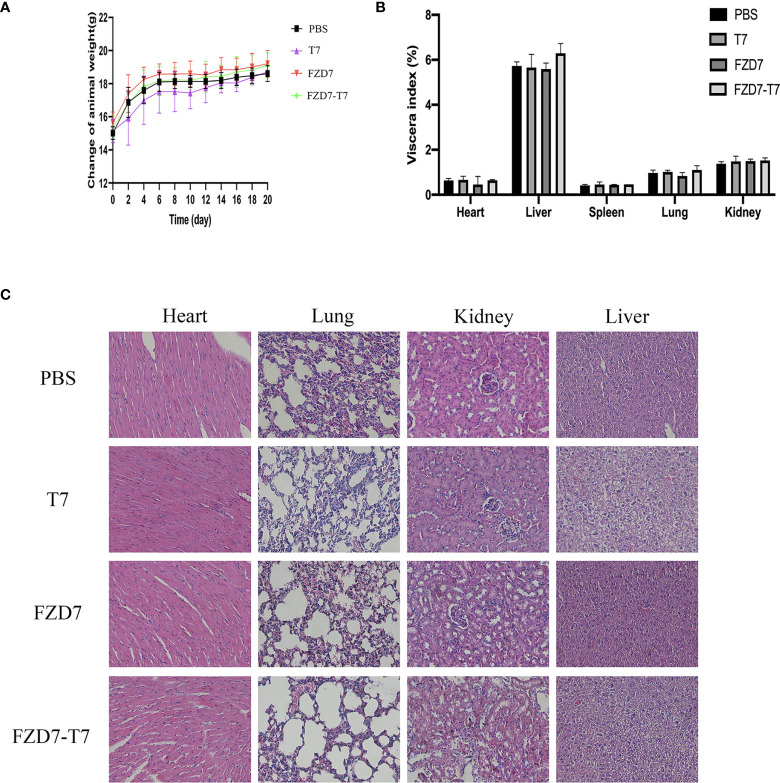
Toxicity studies. **(A)** Bodyweight curve of mice during immunization. **(B)** Viscera index of mice after immunization. **(C)** HE staining of mouse viscera.

Bodyweight curve and organ coefficient are two indispensable indexes in scientific experiments. It can indicate the effect of vaccines on the health status of experimental animals. To observe the effects of immunization on the growth and development of mice, the bodyweight of mice was recorded every two days. There was no statistical difference in body weight between each group and the control group (**Figure 8A**). In 20 days, the mice were weighed and executed in mice after taking out the main organs (heart, liver, lung, kidney, spleen) and weighing, calculating each mouse viscera coefficient (**Figure 8B**) and compared with the control group, each group of mice heart, liver, lung, kidney, spleen, and viscera coefficient, there is no obvious difference, show that there are no obvious immune organs in mice after tissue edema or atrophy of pathological changes (**Figure 8C**).

## Discussion

Tumor cells vary from foreign germs in that their specific protein immunogenicity is low (22), the process of tumor immune evasion is complex, and the immune system has a hard time recognizing them (23, 24). To accomplish the tumor immune killing effect, a viable technique is to locate suitable tumor-specific peptide epitopes and activate the immune response using adequate immune adjuvants (25, 26). The high expression of the FZD7 gene in breast cancer was confirmed in this study, which used BC-GenexMiner V4.3 software to assess the expression of numerous clinical factors of the FZD7 gene in breast cancer patients. The IEDB (http://www.iedb.org/) software was used to predict epitopes and synthesize peptide vaccines from homologous mouse and human FZD7 sequences (27). Small molecular weight, facile modification, simple production, high activity, and minimal toxicity are all advantages of polypeptide drugs (28, 29). We screened the protein antigen epitope polypeptide to manufacture polypeptide medications and found its immunogenicity by an *in vitro* test using contemporary analysis methods (30). *In vivo* studies were carried out to see if the anticancer action could be detected by CD4+ and CD8+ T cell receptors to generate and activate polypeptide-specific tumor-responding T cells *in vivo* (31). After the efficacy of the polypeptide vaccine has been confirmed, the data can be changed as quickly as possible (32).

Several techniques have been designed to suppress Wnt/-catenin signaling by directly or indirectly targeting the FZD7 protein, according to the present literature (12). The soluble FZD7 peptide (sFZD7), for example, prevents Wnt proteins from interacting with THE CRD of FZD7 (33). Anti-FZD7 antibody (FZD7 Ab) prevents Wnt protein from interacting with FZD7 (15). Small molecule inhibitors FJ9 (34) and small interfering peptide (RHPD) disrupt the interaction between the C-terminal tail of FZD7 and the PDZ domain of Dvl. Stable transfection of HCT-116 cells containing FZD7 siRNA reduced *in vivo* metastasis activity (35). The reaction of T cells to FZD7 polypeptide antigen, the clustering of CD4+ T cells/CD8+ T cells (36), and the reaction of immunoglobulin G to vaccination polypeptide were all evaluated in our experiment to develop antibodies against FZD7. The protein FZD7 can be targeted and inhibited. As a result of inhibiting the Wnt signaling system, cell viability, migration, and invasion were reduced. Our experimental results on the therapeutic effect of polypeptide combination vaccine on 4T1 tumors were essentially consistent with the results presented above, indicating that the FZD7-T7 polypeptide vaccine can target and eradicate cancers with high FZD7 protein expression.

Although some research has shown that FZD7 shRNA can reduce TNBC cell proliferation (9), viral therapy can cause unfavorable immunological responses, and adenovirus-mediated cancer gene therapy is still being investigated. Cancer vaccines made up of polypeptides are generally well tolerated and have few side effects. Only 1.2 percent of patients vaccinated with polypeptide vaccines reported vaccine-related major side effects, according to a meta-analysis of 500 patients (37). We performed blood routine, blood biochemistry, organ index, and organ section on immunized mice in this experiment, and found that the aforementioned indexes did not change considerably in the four groups of mice, and the values were within the normal reference range, with little harmful and adverse effects.

The advancement of this topic will help us gain a better understanding of the tumor-suppressing impact and mechanism of peptide vaccines, as well as offer the essential experimental foundation for tumor vaccine application (38). This study used preparation and injection to assess the anticancer efficacy and biosafety of synthetic peptides, as well as techniques for including peptide vaccines in therapeutic immunization. In general, it has a bright future in the tailored treatment of tumors. One of the most potent anticancer therapeutic techniques in the future is antigen-specific T cell immunotherapy, which includes therapeutic peptide immunotherapy (39).

## Conclusions

Studies on anticancer vaccinations based on the FZD7 antigen have clarified the effects and revealed the mechanisms. The FZD7-T7 vaccine has been shown in studies to diminish tumor growth and greatly extend survival time. Vaccines may work by inducing DC cell maturation and activating T cell responses, which is followed by increased levels of lymph cytokines and antibodies to IL-2, IL-12, and IFN-γ. The vaccine was found to improve both humoral and cellular immunity. Furthermore, the vaccine can efficiently cause tumor cell death and regulate the M2 to M1 transformation of tumor-infiltrating macrophages. Finally, toxicity tests demonstrated that the vaccine was safe, providing proof for further use.

## Data availability statement

The raw data supporting the conclusions of this article will be made available by the authors, without undue reservation.

## Ethics statement

The animal study was reviewed and approved by the Laboratory Animal Ethics Committee of Shenzhen University.

## Author contributions

ZH, YH, KL, and HY performed the experiments and analyzed the data. CZ, SN, and FC provided clinical specimens. ZH, YW, ML, and QY wrote the manuscript and supervised the study. CW and XW designed or/and supervised this project and revised the manuscript. The authors read and approved the final manuscript.

## Funding

This work was supported by the National Natural Science Foundation of China (NSFC) (81772002), Shenzhen Subject Layout Project (No. JCYJ20170818092553608), Shenzhen Basic Research Project (No. JCYJ20170303160906960), and Shenzhen Science and Technology Innovation Commission (No. GJHZ20170313111237888).

## Acknowledgments

We thank the laboratory members for their help and support.

## Conflict of interest

The authors declare that the research was conducted in the absence of any commercial or financial relationships that could be construed as a potential conflict of interest.

## Publisher’s note

All claims expressed in this article are solely those of the authors and do not necessarily represent those of their affiliated organizations, or those of the publisher, the editors and the reviewers. Any product that may be evaluated in this article, or claim that may be made by its manufacturer, is not guaranteed or endorsed by the publisher.
